# A systematic review on the eating behaviors of youth exceeding electronic device recommendations

**DOI:** 10.3389/fpsyg.2025.1649571

**Published:** 2025-09-19

**Authors:** Anna Coleman, Devin Neumann, Angela Sasaki Cole, Clark Brady, Alex Park, Jason Odisho, Kaylynn Moschke, Logan Nauts

**Affiliations:** Central Michigan University College of Medicine, Mount Pleasant, MI, United States

**Keywords:** youth, adolescent, screen use, eating disorders, television

## Abstract

**Background:**

As the rates of both disordered eating and electronic device usage in youth and adolescents have increased over the last two decades, several studies have looked to determine if there is any relationship between the two variables. While excessive screen use has broadly been shown to correlated with abnormal eating behaviors, newer research reveals that different types of screen use (e.g., social media, television, or video games) affect eating behaviors in youth and adolescents. The goal of this systematic review is to analyze to what extent different types of screen use are associated with disordered eating habits, as well as assess for nuance in both the age of the population studied, as well as the geographic location of the study.

**Methods:**

A search of relevant terms was conducted from PubMed (*n* = 1,234) and Scopus (*n* = 301) in July of 2024. Included articles examined the relationship between one or more types of screen use and eating behaviors or disorders, were published between 2014 and 2024, and involved participants aged 0–17. Exclusion criteria consisted of articles solely looking at BMI and/or diet as an outcome, as well as articles that studied an adult population. Risk of bias was assessed using the MMAT® 2018 criteria.

**Results:**

Sixteen studies were included in the final analysis, majorly consisting of cross-sectional studies. Results indicated that while there is overwhelming evidence that excessive screen use is correlated with disordered eating habits at large, individual trends in disordered eating vary depending on the type of screen exposure. Furthermore, some studies suggest that disordered eating is a secondary sequela associated with screen time, with the primary cause being lack of sleep or poor mental health.

**Discussion:**

This review is limited by small number of included studies, high number of cross-sectional studies, and small number of studies looking at populations under age twelve. These findings provide opportunities for both clinicians to evaluate their pediatric patients more holistically when treating for disordered eating behaviors. Furthermore, both clinicians and public health officials alike should consider type of screen time when making recommendations for healthy amounts of screen time for children and adolescents.

## 1 Introduction

Screen-based technologies encompass multiple platforms, including television, social media, smartphones/tablets, computers, and video games. Within recent years, the use of screen-based technology has rapidly increased. The American Academy of Pediatrics (AAP) publishes guidelines regarding a “safe” amount of screen exposure for different age groups, although many children consume screen-based media in higher amounts than these guidelines suggest (American Academy of Pediatrics, 2025; Hong et al., 2018). This increased media consumption is associated with a multitude of negative health outcomes including myopia, obesity, cognitive delay, behavioral problems, and decreased motor skills (Ha et al., 2025; Haghjoo et al., 2022; McArthur et al., 2022; Liu et al., 2022).

At the same time, both eating disorder diagnoses and disordered eating habits in youth and adolescents have increased exponentially as well (Galmiche et al., 2019). “Disordered eating habits” can be defined as irregular eating behaviors that do not qualify as an eating disorder per the DSM-5 criteria. A 2023 Journal of American Medicine meta-analysis found a prevalence of 22% for disordered eating habits in those 6–18 years old across sixteen different countries, with the highest concentration found in older adolescents, females, and those with a higher BMI (Lopez-Gill et al., 2023).

A multitude of studies suggest a positive relationship between screen-based technology and disordered eating. One proposed mechanism is that increased exposure to idealized body types and diet-centric content on social media platforms may contribute to a heightened risk for disordered eating via internalization of thin ideals (Blackburn and Hogg, 2024; Rodgers et al., 2019). Social media exposes youth and adolescents to idealized body images, which has the potential to poster unhealthy and/or disordered eating habits in order to obtain this idealized body standard. Additionally, passive screen use, such as television, promotes sedentary lifestyles and mindless eating patterns, disrupting normal hunger cues and contributing to abnormal eating behaviors (Pearson et al., 2017). Finally, the use of screen-based technology in general often replaces time spent in activities that normally protect against disordered eating, particularly physical activity and in-person peer interactions. The loss of these protective behaviors can increase an individual’s probability of developing a disordered eating pattern.

The current literature suggests that different *types* of screen exposure affect eating behavior differently, with some types of screen exposure shown to correlate with disordered eating more often than others. Despite this, there has not yet to date been a systematic review that evaluates this new research in a clinical context. This review aims to evaluate the current literature regarding the impact of excess screen use on the eating behaviors of children and adolescents worldwide, with the goal of providing synthesized analysis of existing knowledge in order to inform the development of disordered eating prevention strategies and identify gaps in current knowledge. Secondary goals of this review are to identify potential nuances between type of screen use and disordered eating habits in children of different ages, as well as children living in different geographic regions. A better understanding of the interaction between screen-based technology and disordered eating is critical to understand and combat the worldwide rise in disordered eating habits.

## 2 Methods

### 2.1 Research question

**Figure 1:**
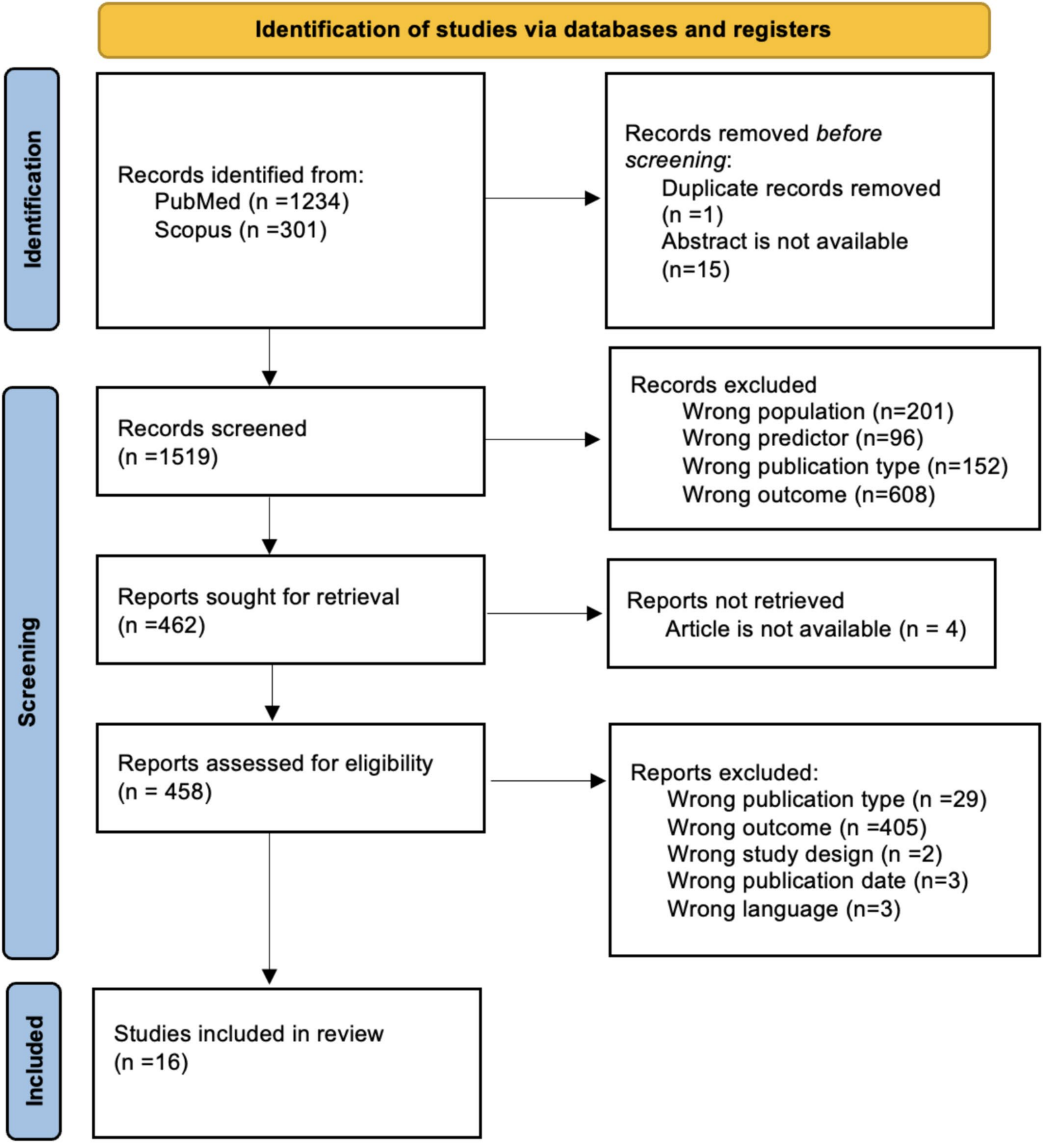
PRISMA diagram.

The research question was developed in accordance with the Population, Intervention, Comparison, Outcome, and Settings (PICOS) criteria (Page et al., 2021) (Table 1). This systematic review was conducted in accordance with the Preferred Reporting Items for Systematic Reviews and Meta-Analyses (PRISMA) 2020 guidelines (Figure 1).

**Table 1:** PICOS criteria.

Parameter	Inclusion criteria
Population	Children aged 0–17
Intervention	Excessive screen time
Comparison	Pediatrician guidelines or to a control group not receiving as much screen time
Outcome	Disordered eating habits or diagnosed eating disorders
Setting	Worldwide

### 2.2 Search strategy

The original systematic review search was conducted in June of 2024 using PubMed® and Scopus®. These databases were selected due to their size and availability through our institution. Combinations of keywords and Medical Subject Headings (MeSH) terms were generated with the help of a medical librarian. The keywords that were used were “technology” and “youth.” A full list of MeSH terms can be found in Box 1.

**BOX 1 Search terms used**

“television” [Mesh] OR “video games” [Mesh] OR internet [Mesh] OR “social media” OR “social networking” OR “cell phone” OR “smartphone” OR “computer” AND “eating disorders” OR “anorexia nervosa” OR “builimia nervosa” OR “binge eating” OR “binge eating disorder” OR “eating behavior” OR “eating habits” AND “adolescent” OR “child” OR “pediatric”

### 2.3 Inclusion and exclusion criteria

Studies were included in this systematic review if they were published within 10 years of the literature search (between 2014 and 2024), isolated a pediatric population defined as between the ages of 0–17 at all points of data collection, and studied the relationship between an excess of one or multiple types of screen exposure and eating behaviors or disorders. “Excess screen use” is defined as screen use either exceeding AAP recommended guidelines, or a higher amount of screen use compared to a control group using a lower amount of screen use. Studies were included regardless of geographic population studied.

Studies were excluded if the sole outcome was BMI and/or obesity or dietary intake (e.g., fruit and vegetable vs. junk food). Though these outcomes can still reflect aspects of disordered eating habits, these studies did not report disordered eating habits or behaviors as a primary outcome. Exceptions were made if an article had an outcome of *excess* junk food (e.g., snacking), as this suggests that the individuals were eating beyond their natural hunger cues, therefore suggesting a disordered eating pattern. Additionally, in order to ensure that all studies only assessed the impact of screen use on a pediatric population, studies were also excluded if part of their sample included 12th graders and/or 18-year-olds. Finally, studies were excluded if they reported screen time as an outcome of disordered eating, rather than as screen time as a predictor of a disordered eating outcome.

### 2.4 Study selection and data extraction

The article search generated 1,605 abstracts. Abstracts were stored in Rayyan®, an online citation manager designed for systematic reviews. Screening was completed by all authors, with each abstract reviewed by at least two authors, in order to ensure accuracy and inter-rater reliability. Decision conflicts were resolved between the individual authors assigned to screen each abstract. If necessary, conflicts were resolved by the first author. After abstract screening, full-article screening and data extraction were done concurrently. Each author was included in this process. The extracted data included: final inclusion or exclusion decision criteria, predictors, outcomes, age group, study population, study design, sample size, significance of findings, and primary conclusions reported by the author. Sixteen articles were included after the full article review stage (Table 2).

**Table 2:** Included studies.

Authors (Year)	Age group	Location	Type of screen use	Findings
Nagata et al. (2021)	Age 9–11	United States	Several different types	Each additional hour of screen time, especially texting and TV, increased the odds of binge eating disorder
Mougharbel et al. (2020)	Adolescents	Canada	TV, smartphones and computers	There was a significant association between TV and BMI but not between the other types of screen time, nor between TV and eating disorders
Chu et al. (2024)	9–14	United States	General screen time; social media	Each additional hour of both screen time and social media use was associated with higher odds of fear of weight gain, self-worth tied to weight, and binge eating
Peat et al. (2015)	12–17	China	Internet use	Internet access was not significantly associated with a subjective belief of fatness or worry over losing control of eating
Ferguson et al. (2014)	10–17; females	United States	TV and social media	Neither TV nor social media was associated with eating disorder symptoms
Livet et al. (2022)	13–17	Canada	Social media and TV	Higher levels of both social media and TV use were related to a higher incidence of eating related symptoms
Cha et al. (2018)	8th graders and 11th graders	Texas	General screen use	Screen use of greater than six hours per day was associated with increased unhealthy eating and nighttime eating as well as loss of sleep
Delfino et al. (2018)	10–17	Brazil	TV, PC, mobile phones	High screen time, especially TV, is associated with a 44% increase in snack consumption
Fletcher et al. (2018)	16–17	Australia	TV, computers, and video games	Screen use of all types is associated with an increase in snack consumption
Kamaleddine et al. (2022)	3–7	Lebanon	General screen use	A screen time of more than 2 h in children was associated with excessive eating
Kristo et al. (2021)	2–5	Istanbul	TV, smartphones, and computers	There was a significant association between children’s eating habits and TV, tablets, and smartphones, but not with computer use
Saat et al. (2023)	13–17	Malaysia	Several different types	Results varied with the strongest significance between television and laptop use and abnormal eating behaviors
Jeong et al. (2024)	1st graders and 11th graders	Korea	Mukbang and cookbang	Exposure to these videos was correlated with eating quickly
Totland et al. (2017)	9–14	Europe	TV	TV viewing was associated with irregular breakfast habits
Yazici Cakiroglu and Sapsaglam (2024)	3–5	Turkey	Overall screen time	Children with higher screen use, especially over 4 h per day, showed more negative eating behaviors compared to those with lower screen use (<1 h per day)
Ural et al. (2024)	4–7	Turkey	Video games	Digital game addiction had a weakly positive correlation with food responsiveness, emotional overeating, slowness in eating, and emotional undereating. It was weakly negatively correlated with enjoyment of food.

### 2.5 Assessment of risk of bias

The “Mixed Methods Appraisal Tool” (MMAT 2018) was used to evaluate the risk of bias in each study included in this systematic review. This tool was selected as it is designed to assess the risk of bias in studies with different experimental designs. The MMAT identifies a list of five criteria for each type of study, thus a score of 0–5 was given to each article included in this systematic review. If a study met all five criteria, the risk of bias was deemed “very low,” four was deemed “low,” three was deemed “medium,” two was deemed “high” and one or zero was deemed “very high” (Table 3). Evidence levels were assigned based on a quality rating system adapted from the Oxford Centre for Evidence-Based Medicine (OCEBM Levels of Evidence Working Group, 2025). By evaluating both the risk of bias and the strength of evidence, the authors were able to conduct a comprehensive evaluation on the quality of each study.

**Table 3:** Risk of bias analysis.

Paper	Study design	Risk of bias	Evidence level
Nagata et al. (2021)	Prospective cohort	Very low	2
Mougharbel et al. (2020)	Longitudinal	Very low	3
Chu et al. (2024)	Prospective cohort	Very low	2
Peat et al. (2015)	Cross sectional	Low	4
Ferguson et al. (2014)	Cross sectional	Low	4
Livet et al. (2022)	Longitudinal	Low	3
Cha et al. (2018)	Cross sectional	Low	4
Delfino et al. (2018)	Cross sectional	Low	4
Fletcher et al. (2018)	Cross sectional	Very low	4
Kamaleddine et al. (2022)	Cross sectional	Low	4
Kristo et al. (2021)	Cross sectional	Low	4
Saat et al. (2023)	Cross sectional	Very low	4
Jeong et al. (2024)	Cross sectional	Very low	4
Totland et al. (2017)	Cross sectional	Low	4
Yazici Cakiroglu and Sapsaglam (2024)	Cross sectional	Very low	4
Ural et al. (2024)	Cross sectional	Very low	4

## 3 Results

After the final article screening process, a total of sixteen studies met the inclusion criteria. The findings of these studies can be categorized into three main classes: results regarding eating disorders, results regarding excessive eating and snack consumption, and results regarding irregular eating habits.

### 3.1 Eating disorders

Eight of the included studies looked at the relationship between prolonged screen use and eating disorders. Of these, five of the studies found a statistically significant correlation between prolonged screen use and increased disordered eating behaviors.

Nagata et al. (2021) found that increases in television, texting, and social networking in preteens (age 9–10 at baseline, then 10–11 at follow-up) in the United States were significantly associated with an increase in the incidence of binge-eating disorder. However, video games and video chat had no significant effect. A cross-sectional study performed by researchers Saat et al. (2023) evaluated disordered eating in Malaysian adolescents as the sum of three categories: emotional eating, external (excess eating), and restrained (restricted) eating. Saat et al. (2023) found a variety of results: excess desktop computer and non-handheld game console use was significantly correlated with a higher disordered eating score on the weekdays, while excess television and laptop use was significantly correlated with a higher disordered eating score on the weekends. Researchers Chu et al. (2024) examined whether there was a difference in the relationship between excess overall screen time and eating disorder symptoms in a prospective cohort study of individuals aged 9–14 throughout the United States. Chu et al. (2024) found that for both screen time and social media use, each additional hour of exposure was correlated to higher odds of eating disorder symptoms, including fear of weight gain, self-worth tied to weight, compensatory behaviors to prevent weight gain, and binge eating. A longitudinal study of Canadian adolescents by Livet et al. (2022) found that both excess television and social media use were more associated with symptoms of eating disorders. Finally, researchers Ural et al. (2024) examined children aged 4–7 in Turkey who were clinically addicted to digital video games (computer games), finding that “digital game addiction” had a weakly positive correlation with both emotional overeating and emotional undereating.

Three of the studies did not find a significant association between excess screen exposure and eating disorder onset or behaviors. A longitudinal study performed by researchers Mougharbel et al. (2020) examined the relationship between television use and eating disorders in Canadian adolescents and did not find a significant correlation. Similarly, in a cross-sectional study of females aged 10–17 in the United States, researchers Ferguson et al. (2014) found that neither increases in television exposure nor social media exposure correlated with eating disorder symptoms. Finally, Peat et al. (2015) found that in Chinese adolescents, increases in internet use was not significantly associated with a subjective belief of fatness or worry over losing control of eating.

### 3.2 Excessive eating/snack consumption

Five of the included studies analyzed the relationship between excess screen exposure and excess eating, either in the form of persistent snacking or consuming extra meals throughout the day. All five studies found a significant relationship between the two variables. Researchers Delfino et al. (2018) examined associations between excess screen time and snack consumption in Brazilian individuals aged 10–17 and found that both television viewing and mobile phone use were linked to significant increases in snack consumption. A similar study performed by researchers Fletcher et al. (2018) found that total screen time was correlated to an increase in snack consumption in Australian 16 and 17-year-olds. In a cross-sectional study performed in Lebanon, researchers Kamaleddine et al. (2022) found that total screen time exceeding guidelines was associated with an increase in excessive eating, particularly of unhealthy foods, in children aged 3–7. In a cross-sectional study examining 8th and 11th graders (age 13–14 and 16–17) in Texas, researchers Cha et al. (2018) found that excess screen time was significantly associated with excessive eating and nighttime snacking. Finally, researchers Yazici Cakiroglu and Sapsaglam (2024) found that in young children in Turkey, higher screen use was associated with more negative eating behaviors, including increases in snack consumption.

### 3.3 Irregular eating behaviors

The remaining three included studies examined the relationship between excess screen time and irregular eating behaviors, defined as an irregular eating pattern that differs from an increase in food consumption. All three studies found a significant relationship between the two variables. Two cross-sectional studies, one of children aged 2–5 in Istanbul published by researchers Kristo et al. (2021) and one of children aged 9–14 across Europe published by researchers Totland et al. (2017) examined the relationship between excess screen time and skipping breakfast. Kristo et al. (2021) found that television and smartphone or tablet use was significantly associated with skipping breakfast, but computer use was not. Similarly, Totland et al. (2017) found that television use was significantly associated with skipping breakfast. Jeong et al. (2024) performed a large cross-sectional study of Korean youth exposed to mukbang and cookbang, genres of Korean television where individuals eat large amounts of food very quickly, finding that increased exposure led to faster eating in children.

### 3.4 Assessment of risk of bias

The risk of bias for each article was assessed using the MMAT® tool (details in the methods section). Eight of the articles received a risk of bias score of “low,” and the other eight received a risk of bias score of “very low.” The majority of the studies (*n* = 12) were cross-sectional and were assigned an evidence level 4. These studies are limited in that they majorly include self-reported or parent-reported data, introducing potential measurement bias. Cross-sectional studies are also limited in their ability to establish causality.

## 4 Discussion

The major finding of this systematic review is that excess electronic device use is linked to both disordered eating behaviors and eating disorders in pediatric populations worldwide.

The electronic device modalities that were shown to have the greatest correlation with an increase in eating disorders were television (Nagata et al., 2021; Livet et al., 2022) and social media (Nagata et al., 2021; Chu et al., 2024; Livet et al., 2022). These modalities were shown to have a positive correlation with several different types of eating disorders, including both the binging and restricting subtypes. One potential explanation for this phenomenon is that the television and social media use could potentially decrease the overall self-esteem in adolescents who use them. Low self-esteem can contribute to binge eating disorders, as food may provide comfort or emotional relief (Pearl et al., 2014), and it can contribute to restrictive eating disorders through over idealizing thin body standards, as teens in this category believe that their self-worth is tied to their thinness (Kowalewska and Mazur, 2023; Button et al., 2003). Similarly, the use of social media has been linked to perfectionism (Gladkaya et al., 2024), a personality trait which also has demonstrated ties to both binging (Halmi et al., 2000) and restrictive eating disorders (Flett et al., 2011). A potential link between television and perfectionism has not been as thoroughly explored.

A possible connection between these types of screen use and self-esteem or perfectionism could explain why they are more likely to cause disordered eating in adolescent populations than other types of screen use, specifically video games. Nagata et al. (2021) did not find a link between video games and binge eating disorders, and Saat et al. (2023) only found a link between video game use and disordered eating if the device was used during the week instead of the weekend. This aligns with the proposed explanations; the body image ideals presented in television and social media could be pushing adolescents to adopt traits of low self-esteem and/or perfectionism, leading to an increased likelihood of disordered eating. With video games, the adolescent is less likely to be exposed to unhealthy body image ideals, potentially explaining why they are less likely to develop an eating disorder.

Three of the included studies (Mougharbel et al., 2020; Peat et al., 2015; Ferguson et al., 2014) did not report any significant association between screen use and disordered eating. Mougharbel et al. (2020) specifically looked at TV use in Canadian adolescents and assessed disordered eating using the Dutch Eating Behavior Questionnaire, which specifically assesses for emotional, external and restrained eating (van Strien et al., 1986). The two studies that found a significant association between excess television exposure and disordered eating were Nagata et al. (2021), who found that more TV use increased the odds of binge-eating disorder, and Livet et al. (2022), who found that TV use increased symptoms of both restrictive and binge eating. All three of these studies were performed with an adolescent group in a Western country. The contradiction in these findings suggests that there is nuance between exactly which types of eating disorders correlate with an excess of TV exposure, and to what extent. Similarly, Peat et al. (2015) did not find a significant association between internet access and disordered eating symptoms in Chinese adolescents, but it is important to note here that these researchers were comparing a group of adolescents who had the ability to access the internet vs. a group who did not. This differs from studies performed in Western countries, where the question is not whether or not the child has access to screens, but whether the child is using screens beyond published recommendations. Finally, the Ferguson et al. (2014) study also reported null results, finding that neither TV nor social media was associated with eating disorder symptoms in females aged 10–14 in the United States. A potential explanation for this discrepancy is that the sample size only consisted of females, thus cannot be directly compared to the other studies that found a positive association.

This review also found that screen use is linked to an increase in excessive eating and snacking. However, only a study by Delfino et al. (2018) looked at the effects of different types of screen use, finding that both television and cell phone use were positively correlated with snacking. Interestingly, these modalities also showed the highest correlation with disordered eating. It may be reasonable to assume that the same mechanism by which television and cell phone use contribute to disordered eating is also responsible for increased snacking; adolescents could be using excessive food as a “comfort” and “emotional escape” due to low self-esteem. However, this idea is limited in that only one study established this correlation. The other three studies that examined a link between screen time and excessive eating or snacking found a positive correlation, but only looked at screen use collectively (Kamaleddine et al., 2022; Fletcher et al., 2018; Cha et al., 2018).

Furthermore, this review found that television use was associated with irregular eating behaviors, notably skipping breakfast (Kristo et al., 2021; Totland et al., 2017). Researchers Chen et al. (2022) suggesting that individuals who skip breakfast and watch excessive television share genetic markers, which could explain their cooccurrence. In addition, excessive television use could disrupt sleep patterns (Buxton et al., 2015; Cain and Gradisar, 2010), leading to rushed mornings and skipping breakfast. Kristo et al. (2021) also found that smartphones and tablets correlate with skipping breakfast. Smartphone and tablet use can disrupt sleep patterns in the same way that television use can (Pérez-Chada et al., 2023).

Thus, this review demonstrates that the modalities of screen use most associated with disordered eating in adolescents are television and social media, likely due to the negative psychological effects that these modalities produce. However, this review is limited by the lack of studies that evaluate these effects by modality. Many of the studies that met the inclusion criteria only examined the effects of screen use as a collective. Furthermore, this systematic review is limited by publication bias, as studies with significant findings are more likely to be published, therefore eliminating potential studies with null results from the data. Finally, due to the variability across studies in age and screen type, it is hard to draw firm conclusions about how *exactly* different types of screen use affect eating behaviors based on the available data. This review is also limited by measurement bias, as the majority of the studies were cross-sectional and thus dependent on self-reported or parent-reported data.

Another limitation of this review is that most of the included studies examined an adolescent population, limiting this review’s ability to apply findings to younger children, specifically those under age twelve. The lack of literature available in this area is likely because disordered eating patterns are more prevalent in adolescence (Stice et al., 2013), however, the prevalence of disordered eating in younger age groups is increasing (Micali et al., 2015). Thus, there is a need for future studies to evaluate how each common screen modality affects eating behaviors in both younger children and adolescents. This will provide for a more comprehensive understanding of how the increase in screen use is affecting the eating behaviors of the youth population.

While a major strength of this review is the inclusion of studies conducted in a variety of different countries, it is important to point out that a limitation of comparing such studies is that cultural background and socioeconomic status are hard to control for. While these confounding variables were controlled for within some of the individual studies, these variables are too complex to control for in a larger-level analysis. This also limits this review’s ability to establish concrete findings.

Future research directions, in addition to providing new studies that contribute to this body of literature, could include longitudinal designs to examine and evaluate potential mediating variables, including sleep and body image. This would allow researchers to fully determine to what extent disordered eating is directly associated with screen use, and to what extent it is a secondary sequela of a confounding variable. New studies could also be conducted on designing interventions based on these findings to lower rates of disordered eating in youth and adolescents worldwide.

### 4.1 Implications for wearable technology and intervention

Wearable technologies (wearables), such as smartwatches and fitness trackers, offer promising tools to detect and potentially modify the patterns of behavior linked to screen overuse and eating issues in youth. As children’s exposure to digital screens continues to rise throughout the world, wearable devices may serve as critical instruments to observe and intervene in habits that contribute to sedentary lifestyles and unhealthy eating behaviors.

These interventions have been shown to be effective in reducing sedentary behavior. A systematic review found that technology-enhanced programs, including wearable technology, reduced sedentary time by 35–41 min per day in children and adolescents (Stephenson et al., 2017). In another study, a teacher-guided intervention using wearable technology decreased sedentary behavior and increased physical activity in preschool-aged children (Byun et al., 2018). Given the observed links between inactivity, often a product of excess screen time, and irregular eating patterns, wearable technology shows promise in decreasing disordered eating behaviors.

Wearable technology is also able to monitor sleep habits, which is increasingly important as digital media has been shown to disrupt sleep routines among youth (Burnell et al., 2022). Wearable technology can also monitor elevated heart rate (due to stress), which tends to have a bidirectional relationship with poor sleep, especially in adolescence. These factors can affect appetite regulation and increase the risk of emotional eating, thus leading to disordered eating. Thus, wearables may not only identify behavioral precursors to disordered eating, but also help target root causes such as sleep dysregulation and psychosocial stress.

Emerging studies further explore if and how wearable technology can monitor eating behavior directly. Several new wearable prototypes in development have been shown to be able to monitor chewing rates and patterns, promoting mindful eating habits in the user (Idris et al., 2021; Nicholls et al., 2019). This offers a potential benefit in youth who struggle with mindless eating while engaging in media use, particularly television and other types of passive screen use.

Wearable technologies offer a multidimensional approach to identifying and addressing the behavioral consequences of screen use that affect eating behaviors in children and adolescents. These technologies create opportunities for immediate interventions, such as reminders to take movement breaks, reminders to unplug before bedtime, or alerts during meals and snacks to foster mindful eating. These strategies may be especially impactful for children and adolescents who, as shown above, are particularly susceptible to the ways that screen use can influence eating behaviors.

## 5 Conclusion

Results indicate that increases in screen time are linked to disordered eating in adolescent populations worldwide, demonstrating a potential contributing factor to the rise of worldwide eating disorder diagnoses and obesity rates. However, different modalities of screen use are correlated with different outcomes in disordered eating habits in these populations. Currently, the AAP makes screen time recommendations based on age alone, however, these findings suggest that pediatric patients could benefit from their clinician taking type of screen time into account when making these recommendations. Similarly, it is important for clinicians to consider both the type and amount of screen time their pediatric patients are exposed to when evaluating potential causes of disordered eating. While more studies are needed to establish firm links between different types of screen time and disordered eating habits policy recommendations could be updated to include current findings, making more targeted suggestions regarding which types of screen time are more beneficial than others. From both a clinical and a public health prospective, the findings of this review and the findings of future studies could improve pediatric health outcomes worldwide.

## Data Availability

The original contributions presented in the study are included in the article/supplementary material, further inquiries can be directed to the corresponding author.

## Glossary

Disordered eating: abnormal eating habits that do not meet the DSM-V criteria for an eating disorder diagnosis; these are typically less severe than eating disorders
Eating disorders: a clinical diagnosis of disordered eating, either restrictive or binge-patterned, per DSM-V criteria
Screen time/electronic device use: the amount of time individuals spend using electronic media for recreational purposes (e.g., gaming, social media, television, or other entertainment purposes)
Social media: digital platforms (e.g., Facebook, Instagram, TikTok) that allow users to create and share photos and videos, as well as interact with others
Television/TV: includes passive viewing of either shows, movies, or sports from any source (e.g., streaming services, cable.) Does not include video content uploaded to social media.
Video games: interactive electronic games played on a screen, either with or without others
